# Management of Cataract in Patients with Irregular Astigmatism with Regular Central Component by Phacoemulsification Combined with Toric Intraocular Lens Implantation

**DOI:** 10.1155/2020/3520856

**Published:** 2020-04-30

**Authors:** Yi Gao, Zi Ye, Wenqian Chen, Jinglan Li, Xinlin Yan, Zhaohui Li

**Affiliations:** Department of Ophthalmology, The General Hospital of the People's Liberation Army, Beijing 100000, China

## Abstract

**Purpose:**

To evaluate visual acuity (VA) and refractive status in patients with cataract and irregular astigmatism with a regular central component after phacoemulsification with implantation of a toric intraocular lens (IOL).

**Methods:**

Patients with cataract associated with irregular astigmatism with a regular central component were enrolled. All patients underwent phacoemulsification and toric IOL implantation. Postoperative visual acuity, residual astigmatism, toric IOL rotation, higher-order aberration, and objective and subjective visual quality were measured 3 months after surgery.

**Results:**

Twenty-three eyes were included in the study. The logMAR corrected and uncorrected distance visual acuity values were decreased at 3 months postoperatively (*p*  <  0.005). The preoperative average corneal astigmatism and postoperative residual astigmatism were 1.15–6.97 D (1.99 ± 1.26 D) and 0–2.75 D (0.65 ± 0.57 D), respectively. The average IOL rotation was 3.17 ± 2.01°. Some objective indicators of visual quality, including the modulation transfer function (*p*  <  0.05), Strehl ratio (*p*  <  0.005), 100% VA (*p*  <  0.005), 20% VA (*p*  <  0.005), and 9% VA (*p*  <  0.005), were significantly higher than the corresponding preoperative values. The objective scatter index (*p*  <  0.005) was significantly lower than that before surgery. The postoperative VF-14 scale score was 83.99 ± 14.58.

**Conclusion:**

Toric IOL implantation has a good corrective effect on certain specific types of corneal irregular astigmatism with cataract. This effect can be attributed to its ability to correct the regular component of irregular astigmatism. The indications for toric IOL implantation could be expanded to some extent, thereby bringing benefit to more patients.

## 1. Introduction

Cataract is a common cause of visual impairment and one of the main causes of blindness worldwide [1]. Preexisting corneal astigmatism of >1.0 *D* has been reported to be present in 23%–47% of eyes with cataract [2]. Corneal astigmatism is typically regular but may be irregular. Toric intraocular lenses (IOLs) are widely used to treat corneal astigmatism and cataract and are an effective and safe treatment for patients with both conditions [3]. Toric IOL implantation has been reported to have a corrective effect on patients with cataract and regular corneal astigmatism [3, 4]. However, some patients with cataract have irregular astigmatism. Uncorrected astigmatism can not only lead to decreased vision but also a decline in quality of vision and quality of life [5, 6]. Therefore, it is important to be able to minimize residual astigmatism postoperatively.

Irregular astigmatism occurs when the angle between the axis of maximum curvature and minimum curvature is not a right angle or when the curvature of a refractive surface is not axially symmetric, and often cannot be corrected with spectacles [7, 8]. There have been reports of effective outcomes after toric IOL implantation in patients with corneal conditions that are associated with irregular astigmatism, including mild or moderate keratoconus, postkeratoplasty, pellucid marginal degeneration, and postpterygium [9–13]. It is possible that certain types of irregular astigmatism could be corrected by toric IOL implantation. In an effort to identify a method that can improve irregular astigmatism as well as supplement and expand the indications for toric IOL implantation, we performed this study to evaluate the postoperative effect of cataract extraction with toric IOL implantation in patients with irregular astigmatism with a regular central component.

## 2. Patients and Methods

### 2.1. Patient Selection

Twenty-three eyes in 20 patients who underwent phacoemulsification with toric IOL implantation between May 2018 and July 2019 at the Department of Ophthalmology, The General Hospital of the People's Liberation Army, Beijing, China, were enrolled in this prospective clinical observational study. All operations were performed by the same experienced ophthalmic surgeon (ZL).

**Figure 1:**
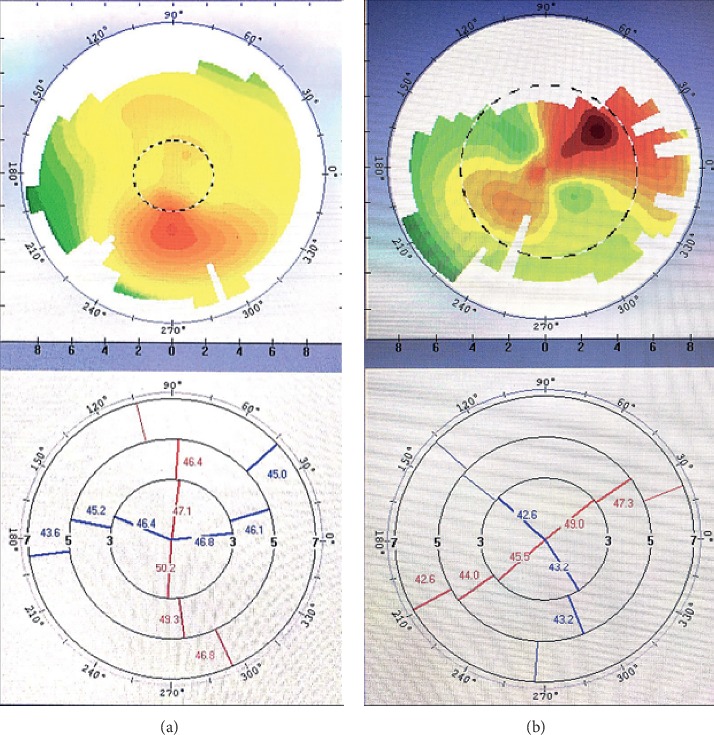
Corneal topography showing a cornea with a hemimeridional pattern. (a, b) The difference in *K* value in the central 3-mm zone between the two hemimeridional lines (in red) was >3 D in each eye.

**Figure 2:**
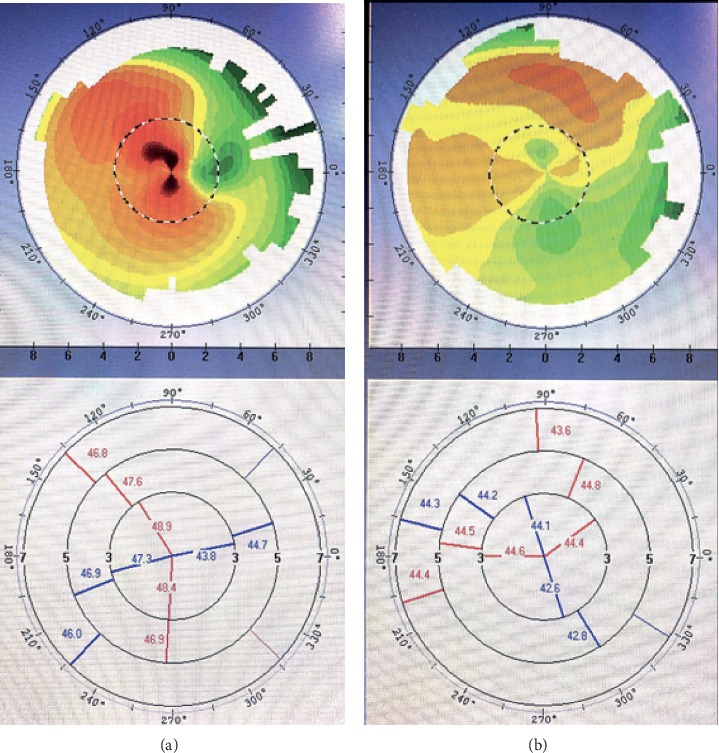
Corneal shape with an inclined hemimeridional pattern. (a, b) Two steep hemimeridional lines (in red) in the central 3-mm zone that do not belong to the same meridian and form an acute angle of 30–45°.

**Figure 3:**
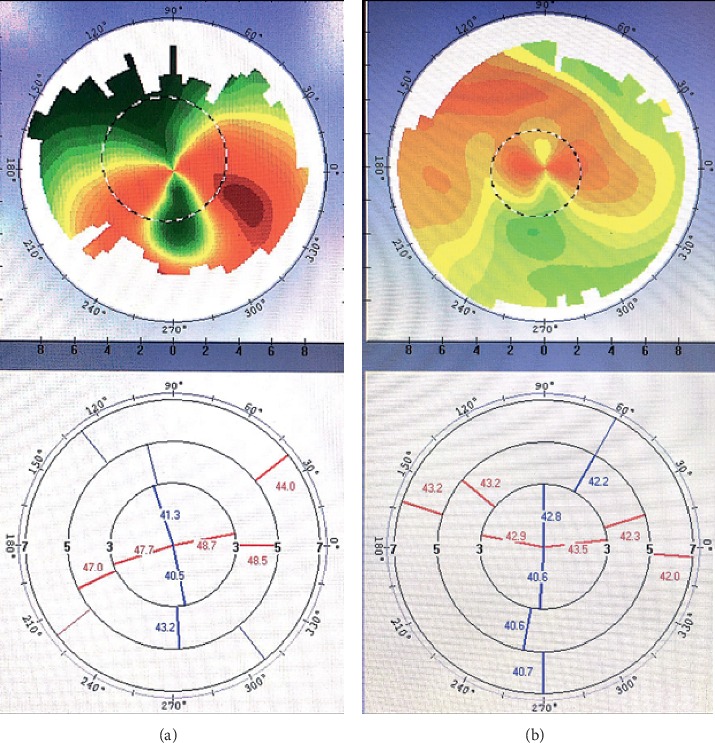
Corneal shape with an irregular periphery pattern. (a, b) Although the two main meridional lines in the central 3-mm zone are in an approximately vertical distribution, an irregular periphery was noted.

The study inclusion criteria were visually significant cataract and astigmatism (>1.00 D). And the type of astigmatism is irregular astigmatism diagnosed by corneal topography [8] and using Bogan's classification [14], and a corneal shape in the central 3-mm zone shows the “bow-tie” pattern on corneal topography. The eyes included were also required to demonstrate at least one of the three following patterns:

- Hemimeridional (two main meridional lines in an approximately vertical distribution in the central 3-mm zone but either unequal slope of the hemimeridians along a single meridian and a difference of >3 D in the *K* value between the two hemimeridians; representative examples are shown in Figures 1(a) and 1(b).)
- Inclined hemimeridional (hemimeridians of an equal slope but not aligned with each other, axially symmetric by another meridional line in the central 3-mm zone, and an acute (30–45°) angle formed by one hemimeridian and extension of the other hemimeridian simultaneously (Figures 2(a) and 2(b) show representative examples).)
- Irregular periphery (two main meridional lines in the central 3-mm zone that show an approximately vertical distribution but an irregular periphery (see Figures 3(a) and 3(b) for representative examples).)

A further inclusion criterion was at least one difference between two axial positions of <10° when measured by three different methods in one eye, i.e., corneal topography, auto keratometry, and the IOL Master.

**Figure 4:**
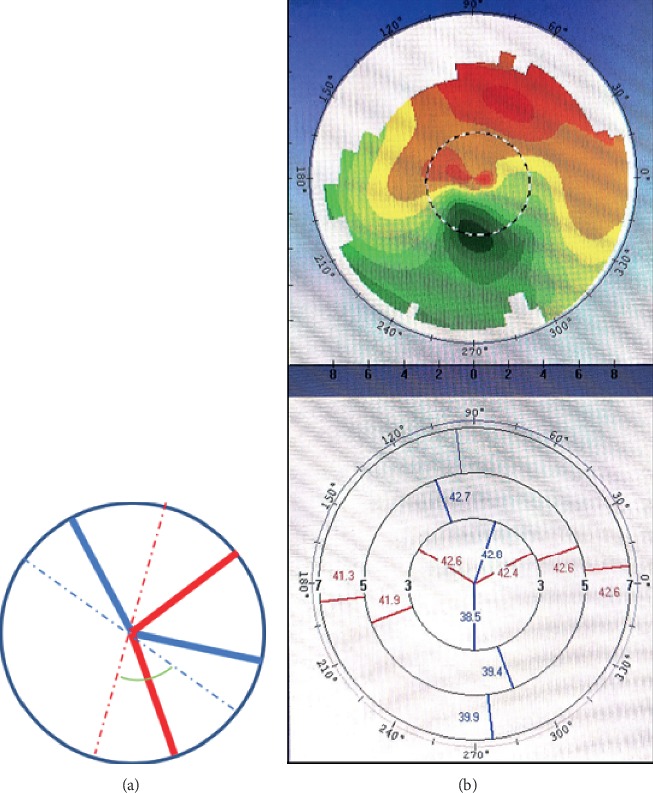
Inclination pattern in the central 3-mm zone. (a, b) show two main meridional lines that are not approximately vertically distributed. The green angle in (a) is obviously not a right angle.

Eyes in which corneal topography showed an inclination pattern of irregular astigmatism, i.e., two main meridional lines that were not approximately vertically distributed (see Figures 4(a) and 4(b) for representative eyes with this pattern) despite an approximately bow-tie pattern being present in the central 3-mm zone were excluded, as were eyes with evidence of other irregular astigmatism patterns, e.g., round, oval, and central irregularity. Furthermore, patients were not enrolled if they had regular astigmatism, progressive corneal disease, dry eye, glaucoma, zonular weakness, uveitis, retinal disease impacting visual acuity (VA), a history of intraocular surgery, and intraoperative complications, such as rupture of the posterior capsule.

All patients provided written informed consent after receiving an explanation of toric IOL implantation and the associated risks.

### 2.2. Preoperative Assessment

All patients underwent a complete preoperative ophthalmic assessment that included slit-lamp examination, measurement of uncorrected distance visual acuity (UDVA) and corrected distance visual acuity (CDVA), phoropter examination using an auto keratorefractometer (KR-800, Topcon Corp., Tokyo, Japan), subjective refraction, applanation tonometry, an endothelial cell count, funduscopy examination, and optical quality analysis (OQAS II, Visiometrics, Barcelona, Spain). Corneal astigmatism and curvature were measured using a Placido-based corneal topographer (WaveLight Allegro Topolyzer Vario, WaveLight GmbH, Erlangen, Germany), KR-8900 auto keratometer, and an IOL Master 700 (Carl Zeiss Meditec AG, Jena, Germany). The average corneal astigmatism (average value of corneal topographer, auto keratometer, and IOL Master 700 values) was calculated. A Pentacam rotating Scheimpflug camera (Oculus, Wetzlar, Germany) was used if needed. Optical biometry measurements, including anterior chamber depth, curvature, and axial length, were obtained by the IOL Master 700 to calculate IOL power.

### 2.3. Toric IOL and IOL Calculation

Only one type of monofocal single-piece toric IOL (AcrySof SN6AT; Alcon Laboratories, Inc., Fort Worth, TX USA) was used during the study period. The SRK-T formula was used to calculate the spherical power of the IOL. The target was defined as emmetropia or −3.0 D myopia depending on the patient' s preoperative refractive status and habit. The cylindrical power of the toric IOL was determined by the online Alcon Barrett toric calculator (http://www.acrysoftoriccalculator.com) using the *K*1 and *K*2 values, axis, axial length, chamber depth, and surgically induced astigmatism (0.3 D). The values for all corneal parameters measured by the three different devices were recorded.The K1(K2) axis value, which is the average of the two more similar values of the three values measured by different devices, was used to calculated the power of IOL.The mean of the *K*1*(K*2 ) values measured by the corresponding two devices was entered into the calculator. Pentacam outcomes were used to assist in decision-making in the event of disagreement.

### 2.4. Surgical Technique

A navigation system (Callisto Eye, Carl Zeiss AG, Dublin, CA) was used during surgery to mark the axis orientation and incision during surgery [15]. All operations were performed by the same experienced surgeon (ZL). Phacoemulsification was performed through a 2.4-mm clear corneal incision. The toric IOL was implanted into a capsular bag, and its position was confirmed as correct using the navigation system.

### 2.5. Postoperative Follow-Up

The patients were followed up 1 day, 1 week, and 1 and 3 months after cataract surgery. UDVA, CDVA, refraction error, and residual astigmatism (obtained from the refraction value) were recorded at each postoperative visit. Three months postoperatively, toric IOL rotation was assessed using a visual quality system (OPD-Scan III, Nidek Inc., Gamagori, Japan) [16]. Higher-order aberration, objective VA, and optical quality were measured by OQAS and a wavefront analyzer (Topcon KR-1W, Topcon Co., Oakland, NJ, USA). All patients completed the Visual Function Questionnaire-14 (VF-14) 3 months after surgery. This questionnaire evaluates visual function using a 0–4-point scoring system (0, unable to complete; 4, no difficulty). The scores for each item are added, averaged, and then multiplied by 25 to obtain the total VF-14 score. The mean VF-14 score for all subjects was recorded.

### 2.6. Data Analysis

VA values were converted to logMAR for analysis. The astigmatism data were analyzed using the power vector method devised by Thibos and Horner [17]. The power vector is a geometric parameter of spherocylindrical refraction with three independent components (*M*, *J*_0_, and *J*_45_). The cylinder power can be decomposed into *J*_0_ and *J*_45_. The formula used to convert these values to power vector coordinates is as follows:

(1) $$\begin{array}{c} M=S+\frac{\left(C\right)}{2},\;\\ J_{0}=-\;\left(\frac{C}{2}\right)*\;\cos\left(2\alpha \right),\\ \;J_{45}=-\;\left(\frac{C}{2}\right)*\;\sin\left(2\alpha \right), \end{array}$$

where *S*, *C*, and *α* represent the spherical power, cylindrical power, and cylinder axis, respectively. *M* is equal to the spherical equivalent.

All patient data were recorded in an Excel database (Microsoft Office for Mac 2016, Microsoft Corp., Redwood, WA, USA). The statistical analyses were performed using SPSS for Mac version 25.0 (IBM Corp., Armonk, NY, USA). All data were first assessed for normality using the Kolmogorov–Smirnov test. The significance of differences in paired data was evaluated using the *t*-test if the data were normally distributed and by the Wilcoxon matched pairs signed-rank test if not. For comparison of different values measured by three different devices, the Friedman test and Bonferroni correction were used. A *p*-value ≤0.05 was considered statistically significant.

## 3. Results

The scheduled follow-up visits were completed for 23 eyes in 20 patients (mean age 71.94 ± 7.76 [range, 53–81] years). The mean preoperative corneal astigmatism measured by the three methods was 1.99 ± 1.26 (range, 1.15–6.97) D. The patient demographics and relevant preoperative data are shown in Table 1.

### 3.1. Visual Acuity

The preoperative and postoperative VA data are shown in Table 2. There were statistically significant improvements in UDVA and CDVA after surgery (*p*  <  0.05). The mean CDVA was 0.46 ± 0.22 logMAR preoperatively and 0.10 ± 0.10 logMAR at 3 months postoperatively; the respective UDVA values were 0.68 ± 0.28 logMAR and 0.22 ± 0.17 logMAR. At the 3-month postoperative follow-up, all eyes had achieved CDVA better than 0.30 logMAR and UDVA better than 0.60 logMAR; the UDVA at this time was 0.3 logMAR or better in 19 eyes (83%) and 0.22 logMAR or better in 15 eyes (65%).

### 3.2. Preoperative Corneal Astigmatism and Residual Astigmatism

**Figure 5:**
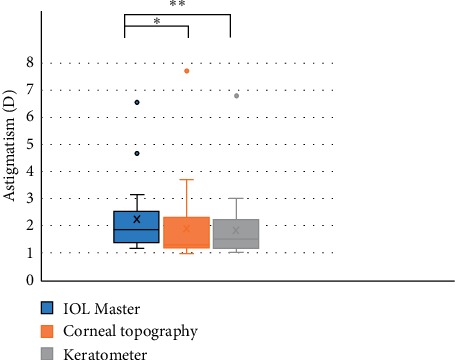
Corneal astigmatism measured by 3 devices preoperatively. The value measured by IOL Master is higher than those by the corneal topography (^*∗*^*p*  <  0.05) and using a keratometer (^*∗∗*^*p*  <  0.005).

**Figure 6:**
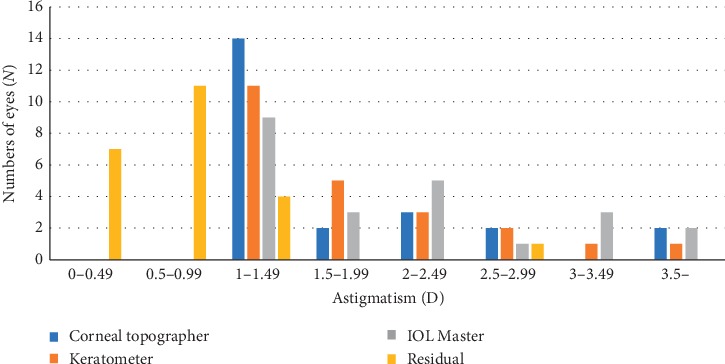
Distribution of corneal astigmatism preoperatively and residual astigmatism 3 months postoperatively.

The preoperative average corneal astigmatism was 1.99  ± 1.26 (1.15–6.97) D, and the residual astigmatism (refractive astigmatism) 3 months after surgery was 0.65 ± 0.57 (0–2.75) D (Table 3). The preoperative corneal astigmatism values measured by IOL Master, corneal topography, and auto keratometer were (2.24 ± 1.25) D, (1.89 ± 1.44) D, and (1.84 ± 1.23) D, respectively. The value measured by IOL Master is higher than those by the corneal topography and keratometer (Figure 5). The distributions of preoperative corneal astigmatism and residual astigmatism are shown in Figure 6. The residual astigmatism was within 0.75 D in 78% of patients and within 1.25 D in 96%.

### 3.3. Rotational Stability

The mean IOL rotation was 3.17 ± 2.01° at 3 months after surgery. No eyes had an IOL rotation of more than 10°. IOL rotation was between 0° and 3° in 16 eyes (70%) and between 3.1° and 6° in 6 eyes (26%). Only one eye had an IOL deviation of more than 6°.

### 3.4. Astigmatism

**Figure 7:**
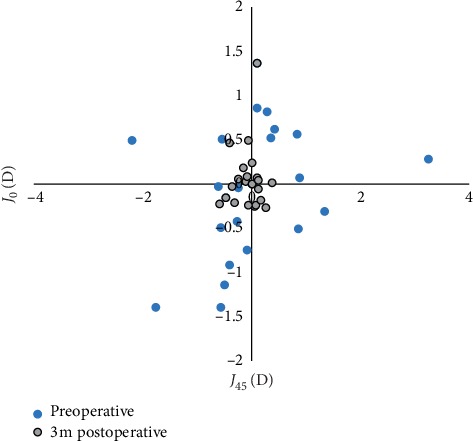
Preoperative and 3 months postoperative coordinate point of astigmatic vectors (*J*_0_, *J*_45_). The dots closer to the origin indicate less astigmatism.

The mean *M* value decreased from −0.46 ± 2.43 to −0.34 ± 1.28 by 3 months after surgery. Postoperatively, the data were located more centrally around the (0, 0) coordinate point (Figure 7). The closer the coordinate point (*J*_0_, *J*_45_) was to the (0, 0) value, the less the astigmatism present. Therefore, there was a significant decrease in astigmatism postoperatively.

### 3.5. Optical Quality

Table 4 shows the preoperative and postoperative optical quality parameters. There were statistically significant postoperative increases in the modulation transfer function (MTF) cutoff value, Strehl ratio, 100% VA, 20% VA, and 9% VA and a postoperative reduction in the objective scatter index (OSI). The mean postoperative higher-order and spherical aberration values measured by the Wave-Front Analyzer were 0.27 ± 0.14 and 0.040 ± 0.094, respectively.

The mean VF-14 score for subjective visual quality was 84.99 ± 13.29 after surgery. The closer the score obtained was to 100, the easier patients found it to perform their activities of daily living. Most patients (87%) were satisfied with the outcome.

## 4. Discussion

At present, toric IOL implantation is widely used to correct regular corneal astigmatism in order to obtain better postoperative visual and refractive effects while removing cataract [3, 4, 18]. Various toric IOLs are now available as a result of technological advances in the materials used in IOLs and their design, which have improved the safety and predictability of these devices [3, 19]. The efficacy and rotational stability of the AcrySof SN6AT toric IOL used in our study is well established [20]. The efficacy and stability of toric IOL implantation when used to treat cataract in patients with certain specific types of irregular astigmatism were evaluated in this study. Our results are similar to those of previous studies that have used toric IOLs to correct irregular astigmatism in keratoconus, postkeratoplasty, pellucid marginal degeneration, and postpterygium [9–13, 21, 22]. In addition to the abovementioned corneal disorders, we also included eyes with old corneal scars but stable astigmatism. Our results suggest that patients with stable corneal pathology and irregular astigmatism with a regular central component can be considered for toric IOL implantation.

In our study, UDVA and CDVA were both significantly improved 3 months after surgery, with a 3-month postoperative UDVA of 20/40 (logMAR 0.3) or better in 19 eyes (83%). Our outcomes are similar to those in a previous study of 53 eyes that underwent AcrySof toric IOL implantation; in that study, the UDVA was 20/40 or better in more than 90% of eyes in groups in which IOL cylinder could fully correct the corneal astigmatism [23]. A randomized controlled study by Holland et al. [24] found that 92% of 256 eyes achieved a UDVA of 20/40 or better. It seems that the VA in the previous study, in which the subjects had regular astigmatism, was better than that in our study. This discrepancy suggests a relationship between irregular astigmatism and residual astigmatism.

The decrease in corneal astigmatism from 1.99 ± 1.26 D before surgery to a residual astigmatism of 0.65 ± 0.57 D after surgery in our study indicates successful correction of astigmatism. The residual astigmatism was within 0.75 D in 78% of patients and within 1.25 D in 96%. This outcome is comparable with the outcomes seen in previous studies. Bauer et al. [23] reported residual astigmatism of ≤0.75 D in 74% of eyes and ≤1.00 D in 91% of eyes. Similar data were reported by Holland et al. [24]. There was one outlier (2.75 D) in our residual astigmatism data. However, the preoperative corneal astigmatism was 6.5 D when measured by the IOL Master, 7.66 D when measured by topography (Figure 3(a)), and 6.75 D when measured by keratometry, with a postoperative spherical equivalent of −0.625 D. The biggest correction power on corneal plane of AcrySof Toric IOL is 4.11 D, and only the central regular portion of irregular astigmatism could be corrected in this patient. More than 50% astigmatism was corrected, but residual astigmatism was inevitable. The patient was satisfied with a UDVA of 20/32 and a CDVA of 20/25.

**Figure 8:**
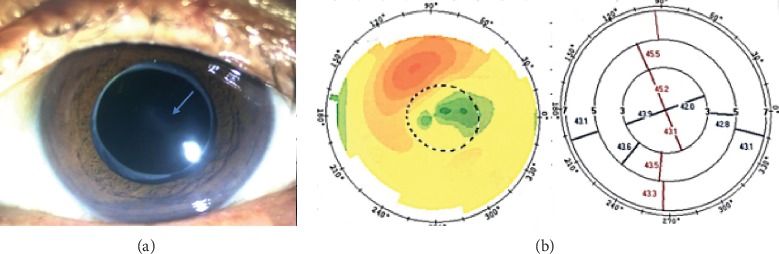
A photo of an eye with an old central traumatic scar and its topography. The scar is indicated by the blue arrow in (a).

One eye with an old central traumatic scar only achieved a UDVA of 20/50 and a CDVA of 20/40 (Figure 8). The flat *K* axes were similar (25°, 20°, and 17.7°) when measured by the three methods, as were the *K* values. The IOL rotation was 6° by 3 months after surgery. However, unexpectedly, the residual astigmatism was 1.25 D with an axis of 85°, which was almost the same as the preoperative corneal astigmatism value. Factors potentially affecting the selection of a toric IOL and resulting in postoperative residual astigmatism errors in this patient include the following: inaccurate measurement of corneal curvature because of ocular surface changes (scars); inappropriate patient selection, i.e., those with highly irregular astigmatism that might affect the results [25]; and errors in the refractive status measured by auto refractometry as a result of corneal scarring [26–30]. Although the preoperative corneal measurements obtained by the different methods were comparable in eyes with central corneal scars, we do not recommend toric IOL implantation in such eyes.

The accuracy and comparability of corneal astigmatism measurements obtained by manual keratometry, auto keratometry, the IOL Master, corneal topography/ray-tracing aberrometry (iTrace), Orbscan, and Pentacam have been demonstrated in eyes with regular astigmatism [31]. However, in some eyes with irregular astigmatism, the keratometric measurements are inaccurate and different from the corneal topography measurements [26]. Roh et al. [27] demonstrated that the classic auto keratometer had a significant influence on the accuracy of assessment of astigmatism in eyes with corneal irregularities. The auto keratometer and corneal topographer have been shown to perform poorly when used to assess corneal astigmatism and axis location in eyes with an irregular corneal surface [28, 29]. Therefore, the eyes with irregular astigmatism enrolled in our study were required to have a reasonably regular central component to reduce errors in measurement. The shape of the cornea is best estimated by corneal topography and the Pentacam [30]. Unlike when using keratometry, the IOL Master, and corneal topography, in which the ratio between the anterior and posterior curvature is assumed to be fixed, posterior corneal astigmatism can be measured directly using the Pentacam. Recent studies have shown that ignoring posterior corneal astigmatism may result in incorrect toric IOL calculation [32, 33]. Koch et al. [34] demonstrated that the with-the-rule astigmatism may be overestimated by 0.5-0.6 D and the against-the-rule astigmatism may be underestimated by 0.2–0.3 D if posterior corneal astigmatism is not considered. There is also a report showing that the axial error was 7.4 ± 10.3° when posterior corneal astigmatism was ignored [35]. Davison and Richard [36] used total corneal astigmatism measured by the Pentacam and spherical power obtained by the IOL Master for AcrySof toric calculations (http://www.acrysoftoriccalculator.com/) and reported a good postoperative refractive effect. Davison indicated that consideration of total astigmatism on an individual basis, rather than using the population-averaged value, provides better results for the patient. The Alcon Barrett toric calculator used in our study compensates for posterior corneal astigmatism [4, 37]. When using this toric calculator, the recommendation is to use anterior surface data instead of total corneal astigmatism to avoid errors. Ferreira et al. [38] demonstrated that direct measurement of total corneal power for toric IOL calculation did not take advantage of the estimation methods available when using the Alcon Barrett toric calculator.

Accurate measurement of astigmatism and axial position before cataract surgery is a prerequisite for accurate calculation of toric IOL power and for achieving an optimal refractive state. Outcomes obtained by different devices may not be consistent because of the different areas measured and different refractive indices used. Measurements may also be affected by the state of the ocular surface and corneal disease [4, 25, 39, 40]. It is essential to use at least two different devices based on different principles for accuracy of corneal outcomes [4, 41]. One report maintained that corneal measurements of axial position and the magnitude of astigmatism should be measured by at least three different instruments. The precision and consistency of measurements are more challenging for irregular astigmatism. Therefore, we use three or even four measurement methods and use the average value for calculation.

Rotational stability is an important concern when performing toric IOL implantation. In one report, each degree of deviation from the predicted axial position led to a 3.3% loss of cylindrical power [42]. Misalignment of the IOL axis by 5° can result in a 7.03% decay in image quality [43]. In our study, the mean IOL rotation was 3.17 ± 2.01° at 3 months after surgery. No eye had an IOL rotation >10°. Rotational stability was good in our study, as reported elsewhere [19].

In this study, we found a significant difference between preoperative and postoperative optical quality. The respective mean postoperative MTF cutoff and OSI values were 21.79 ± 10.62 and 3.13 ± 3.17. The postoperative MTF cutoff value was comparable with that in previous studies, whereas the OSI value was relatively high; the optical quality after toric IOL implantation appeared to be slightly worse in our study [44, 45]. The OSI value was <3 in 17 eyes (74%), and there was one abnormal value of 15.8 in an eye with central corneal scarring and opacity, which underscores the powerful impact on the OSI. Irregular astigmatism may contribute to optical quality. The mean subjective visual quality was evaluated by the VF-14, and the mean score was 83.99 ± 14.58. In total, 87% of patients were satisfied with the outcome of toric IOL implantation.

This study has some limitations. One limitation was the small sample size, which may have resulted in lack of statistical power and introduction of a degree of bias. Another limitation is the lack of a control group of eyes with irregular astigmatism. However, despite these drawbacks, our results are promising. Further studies that include larger sample sizes, carefully selected patients, and longer follow-up durations are needed to confirm our present findings.

In conclusion, phacoemulsification with toric IOL implantation is effective in some patients with specific types of corneal irregular astigmatism accompanied by cataract. Its efficacy can be attributed to its ability to correct the regular component of irregular corneal astigmatism in these patients. Strict patient selection and accurate measurement and calculation would increase the likelihood of this treatment being successful.

## Figures and Tables

**Table 1:** Patient demographics and preoperative characteristics.

Patient (*n*)	20	
Eyes (*n*)	23	
Ages (years)	71.94 ± 7.76	53–81
Average of corneal astigmatism (D)	1.99 ± 1.26	1.15–6.97
IOL sphere (D)	21.54 ± 1.76	18.5–25.5
IOL sphere (D)	2.62 ± 1.23	1.0–4.5

The data are expressed as the mean ± standard deviation (range).

**Table 2:** Preoperative and postoperative visual acuity.

Parameter	Preoperative	Postoperative			
		1 day	1 week	1 month	3 months
UDVA (logMAR)	0.68 ± 0.28	0.25 ± 0.18^*∗∗*^	0.23 ± 0.18^*∗∗*^	0.25 ± 0.18^*∗∗*^	0.22 ± 0.17^*∗∗*^
CDVA (logMAR)	0.46 ± 0.22	0.10 ± 0.10^*∗∗*^	0.09 ± 0.13^*∗∗*^	0.12 ± 0.10^*∗∗*^	0.10 ± 0.10^*∗∗*^

The data are expressed as the mean ± standard deviation. ^*∗*^*p*  <  0.05; ^*∗∗*^*p*  <  0.005 vs the preoperative value.

**Table 3:** Preoperative corneal astigmatism and postoperative residual astigmatism.

	Preoperative corneal astigmatism				Residual
	IOL Master	Keratometer	Corneal topography	Average	
Astigmatism	2.24 ± 1.25^*∗∗*^	1.84 ± 1.23^*∗∗*^	1.89 ± 1.44^*∗∗*^	1.99 ± 1.26^*∗∗*^	0.65 ± 0.57
	1.2 – 6.5	1 – 6.75	1 – 7.66	1.15 – 6.97	0 – 2.75

The data are expressed as the mean ± standard deviation (range). ^*∗*^*p*  <  0.05; ^*∗∗*^*p*  <  0.005 vs. the preoperative value. The average represents average corneal astigmatism (the average of IOL Master, corneal topography, and keratometer values).The residual represents postoperative refractive astigmatism.

**Table 4:** Optical quality values preoperatively and 3 months postoperatively.

Parameter	Preoperative	Postoperative	*p*
OSI	6.89 ± 3.81	3.13 ± 3.17	0.001^b^
MTF cutoff	9.11 ± 4.61	21.79 ± 10.62	0.022^a^
Strehl ratio	0.07 ± 3.81	0.12 ± 0.04	0.001^b^
VA 100%	0.31 ± 0.15	0.72 ± 0.37	0.001^b^
VA 20%	0.19 ± 0.10	0.47 ± 0.24	0.001^b^
VA 9%	0.10 ± 0.07	0.29 ± 0.15	0.001^b^

## Data Availability

The data in this paper are available from the corresponding author upon reasonable request.
